# A Plastic Optical Fiber Sensing System for Bridge Deflection Measurement

**DOI:** 10.3390/s20020480

**Published:** 2020-01-15

**Authors:** Dong Yang, Jin-Qi Wang, Wei-Xin Ren, Jing Zhang

**Affiliations:** School of Civil Engineering, Hefei University of Technology, Hefei 230009, China; yangdong@hfut.edu.cn (D.Y.); jqwang@mail.hfut.edu.cn (J.-Q.W.); zhangj@hfut.edu.cn (J.Z.)

**Keywords:** deflection measurement, connected pipe system, long-span bridge, liquid level sensor, plastic optical fiber sensor

## Abstract

Deflection is one of the key parameters that reflects the state of a bridge. However, deflection measurement is difficult for a bridge that is under operation. Most existing sensors and measuring techniques often do not meet the requirements for health monitoring for various types of bridges. Therefore, based on changes of optical fiber intensity, a novel sensing system using connected pipes to measure bridge deflection in different positions is proposed in this paper. As an absolute reference, the liquid level position along the structure is adopted for the deflection measurement, and an additional external reference to the ground is not needed in this system. The proposed system consists of three parts: connected pipes to connect the measurement points along the structure, liquid to fill in the connected pipes, and the sensing element to detect the change of level. A plastic optical fiber sensor based on the intensity change is used as the sensing element of the developed system. Then, a set of experimental tests are conducted for performance evaluation purposes. Results show that this system has an accurate linear response and high reliability under various environmental conditions. The deflection of the test beam measured by the sensor agrees with linear variable differential transformer (LVDT) within an error margin of 2.1%. The proposed system shows great potential applicability for future health monitoring of long-span bridges.

## 1. Introduction

Structural health monitoring (SHM) is performed to ensure structural operation safety, which involves structural response acquisition, damage-sensitive feature extraction, and structural health state analysis using extracted features. For SHM, proper sensor selection and placement are extremely essential and necessary to determine the quality of collected structural responses, in turn revealing the health condition. Deflection is one of the most important parameters in bridge SHM because it can represent the integral stiffness of the structure, and thus is closely reflected to its load capacity. However, the measurement of bridge deflection is one of the fundamental problems. For bridges, it is difficult to install the existing deflection transducers firmly because most bridges span rivers, sea straits, highways, and mountainous terrain. Therefore, a more convenient, easier to set up, and more affordable deflection monitoring system for bridges has become one of the current urgent requirements.

Over the past few years, a large number of bridge deflection monitoring attempts have been conducted. Dial gauges and linear variable differential transformers (LVDTs) are traditional instruments for direct bridge deflection measurement. Measurement can be achieved by those direct measuring tools in both static and dynamic areas. However, using a fixed platform near or under the measurement points as a reference point is inevitable. The transducers should be installed by connecting the reference point and measurement point of the measuring object. However, the gaps between the reference point and measurement point can be unpredictable if locations of the bridges are uncertain. A temporary scaffold is helpful to solve this problem, but additional apparatuses will cause larger errors and extra costs. Furthermore, those direct measurement technologies are unsuitable when bridges have a large bottom space [1,2]. To overcome these problems, other automatic deflection measurement techniques are usually used for bridges. Bridge deflection measurement can be performed without accessing the structure for either the observation or to connect the platform to the ground [3]. 

Global positioning systems (GPS), acceleration-based methods, strain-based methods, laser technology, radar technology, microwave interferometers, connected pipe systems, and so on are all automatic measurement techniques. Among them, GPS has shown good practicability in recent years. It has been successfully applied to measure the deflection of long-span bridges and the only equipment required is a GPS receptor antenna, which should be installed at the observation points. Good environmental suitability and long-term reliability can be achieved [4,5]. As a developing technology, GPS has practical difficulties in its application. Achievable accuracy is a major barrier, and it is affected by many factors and limitations, such as tracking enough satellites with well-distributed geometries during the lifecycle of a structure. Moreover, the high cost is another practical problem in GPS-based deflection measurement.

For acceleration-based methods, the dynamic response of the deflection can be obtained by double integration of corresponding acceleration responses with appropriate baseline corrections [6]. Faulkner et al. [7] proposed that in order to obtain the bridge displacement time history by numerical integration, the sampling rate is an important factor in the process of acceleration acquisition, and several bridge displacement response calculation methods using measured acceleration data were evaluated. Lee et al. [8] integrated the acceleration data in the frequency domain to estimate the structural deflection. Park et al. [9] developed a low-cost, acceleration-based wireless displacement monitoring system for civil engineering structures. The acceleration-based method is convenient, low-cost, and attractive, but lacks reliability in field testing.

For strain-based displacement estimation, Wang et al. [10] proposed a strain-sensor-based method to estimate the displacement response using the strain mode shapes of beam structures. Glaser et al. [11] determined the mode shape of a beam structure based on corresponding curvature and strain measurements. Numerical simulations and experimental investigations were presented to verify his proposed method. The strain-based methods, similar to the acceleration-based methods, are also sensitive to noise, and complex algorithms are involved in extracting displacement from strain, which make their use difficult for practical applications.

Zhang et al. [12] focused on remote monitoring of vibration displacement responses of two long-span bridges using the Gamma portable radar interferometer, which is a developed multimode microwave interferometric radar system. Liu et al. [13] monitored dynamic responses using ground-based microwave interferometry and then analyzed ancient bridge vibration using the extreme-point symmetric mode decomposition method. However, the mentioned devices are high-cost and the corresponding method is difficult to use for in-site implementation.

Vision-based methods offer a good substitute to bridge deflection measurement. Vicente et al. [14] presented a new laser- and video-based displacement transducer for monitoring of structural displacements and rotations. The proposed system combines laser beams, LED lights, and a digital video camera. This system was proposed for static and slow-varying displacement monitoring. Lydon et al. [15] presented a non-contact, multipoint displacement measurement system based on multiple synchronized wireless cameras, and calculated the displacements using the computer vision technique, providing a valuable insight into the states of bridge structures in operational conditions. Feng et al. [16] developed a subpixel template matching technique based vision sensor system for non-contact measurement of structural displacement. Xu et al. [17] proposed a low-cost, vision-based system for multipoint displacement measurement using a consumer-grade camera for video acquisition and a custom-developed package for video processing. However, there are still lots of problems with this system, such as difficulties with stable camera mounting [18], measurement errors derived from lighting changes [19], and light refraction caused by atmospheric effects, especially for long-range measurements. 

One of the effective methods is a connected pipe system, which is applicable to both short- and long-term structural deflection monitoring by employing the liquid leveling system as a deflection reference. This technology has been developed over more than 40 years and many research studies have illustrated its efficiency [20,21]. Liu et al. [22] discussed a connected pipe system based deflection monitoring and condition evaluation for a suspension bridge in China, and a series of load tests were also conducted to validate the proposed system. Zhu et al. [23] presented the health monitoring system on Dafosi Bridge. The deflections of this bridge were mainly obtained through a communicating optoelectronic pipe sensor, namely the connected pipe system. Ye et al. [24] proposed a liquid level sensing system to monitor the static and dynamic deflections of a bridge. The sensing elements of this system were pressure transmitters, and this system was improved by a step-type pipeline to remove the interference of the inclination angle [25].

This research proposes an improved connected pipe system for bridge deflection monitoring and the plastic optical fiber is used to fabricate the liquid level sensing element in the system. Compared to other sensing techniques for level acquisition, the plastic optical fiber is less expensive and is easy to install and maintain. More importantly, on the application side [26], it is easy to construct and the interrogation is simple and very low-cost. To obtain the deflection from the system, a simple formula related to the variation of the liquid level is also applied.

## 2. Connected Pipe System for Bridge Deflection Monitoring

The connected pipe method consists of the pipe system, liquid, cylinders attached at the structure, and the liquid level sensing element, which is the element used for deflection acquisition. The connected pipe system and sensing element are discussed in Section 2.1 and Section 2.2, respectively. The manufacture of the sensing element is presented in Section 2.3.

### 2.1. Principle of the Connected Pipe System

The connected pipe system is a well-established tool used to perform static structural deflection monitoring. This system is cost-effective, easy to install, precise, and is a reference for free structural vertical displacement monitoring technology, especially for long-span bridges.

**Figure 1:**
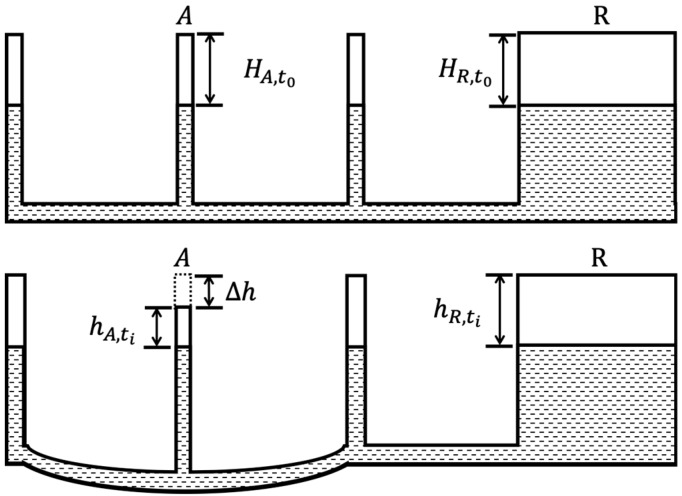
Fundamental principal of the connected pipe system.

In fact, this system is operated by a set of connected pipes and cylinders that are filled with the appropriate amount of liquid, and all connected cylinders are installed to the selected points at the structure for relative vertical displacement measurements. The pipes and cylinders should also be extended to the bridge bearing or any point that can be considered a fixed point. In general, the liquid level will maintain a constant value in every cylinder along the whole system. The vertical deflection of the structure will mean the positions of the cylinders vary, but the liquid level will still stay the same. This will mean the relative height between the liquid level and the cylinders will vary, equaling the vertical displacement of the structure. Moreover, the reading of the liquid level at the reference point should be subtracted from the values of vertical displacement, so that the calibrated vertical displacement can be obtained. The operating principle of the connected pipe system for the structural relative vertical displacement measurement is shown in Figure 1. Here, “A” is the measuring point at a bridge and “R” is the fixed point, which is the bridge bearing in most cases.

The vertical displacement at “A” can be presented as:

(1) $$\Delta h=\left(h_{A,t_{i}}-H_{A,t_{0}}\right)-\left(h_{R,t_{i}}-H_{R,t_{0}}\right)$$

where Δh is the vertical deflection, $h_{A,t_{i}}$ and $H_{A,t_{0}}$ are the distances between the top of the cylinder and the liquid level when the structure is underformed and deformed at point “A”, respectively; $h_{R,t_{i}}$ and $H_{R,t_{0}}$ are the distances between the top of the cylinder and the liquid level when the structure is underformed and deformed at the reference point, respectively. Identically, the vertical displacement of other positions can also be obtained in the same way. In this system, the variants of the liquid level at the reference point and the selected points are all able to be measured by the plastic optical fiber sensing element =. Moreover, according to the variation of the level at the reference point, the state of the connected pipe system can be monitored by the sensor located at the reference point.

In general, the connected pipe system should be efficient and highly accurate, while the sensing element should be applicable if the whole system is installed properly. However, errors are inevitable, which often reduce the accuracy of the system. The common errors of the system and the strategies to eliminate the errors are discussed as follows: (1) The effect of temperature variations at different locations along the whole system is one of the error sources. (2) The air bubbles that exist in the system cause dramatic measurement error. These problems are difficult to avoid during the installation and operation of the system. One of considerations for avoiding errors is to eliminate the bubbles inside the pipe system, and Equation (1) can be helpful for removing temperature variations. Essentially, with careful consideration and installation, it is possible to minimize errors. 

Another problem is the choice of liquid. Water is the most commonly selected liquid, and liquids with various characteristics can be used for different conditions, such as oil for extreme cold conditions because of its low freeze point. When adopting liquids with various densities, the sensitivity of the sensor can also be changed.

### 2.2. Basic Theory of the Liquid Level Sensor

**Figure 2:**
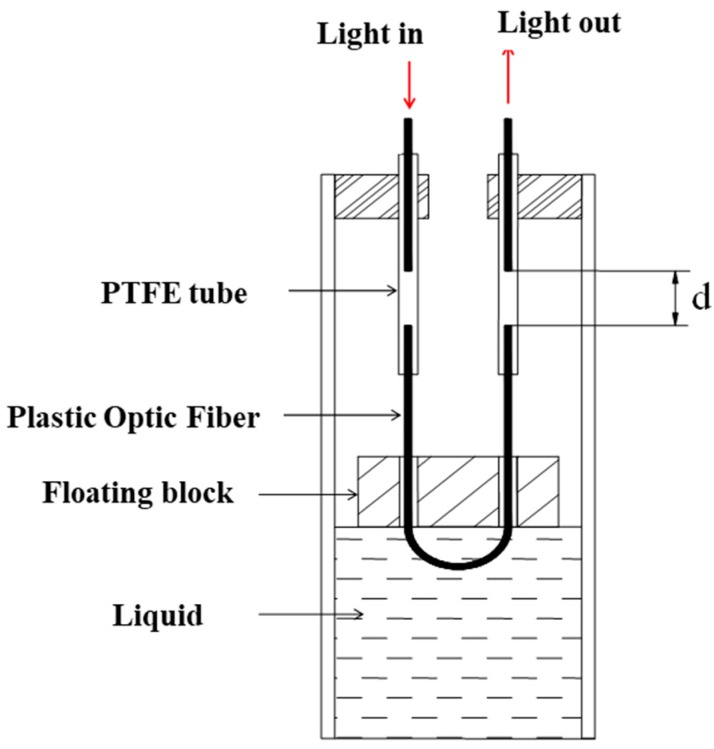
Optical-fiber-based liquid sensor setup. Note: PTFE = polytetrafluoroethylene.

As the sensing element, the liquid level sensor measures the distance variations between the top of the cylinder and the liquid level, which is equivalent to the vertical structural displacement at the selected point. As a substitute for a conventional electrical sensor, the proposed plastic optical fiber sensor (POFS) is simpler, easier to use, and low-cost. In truth, there are several types of fiber optic sensors, some of which are based on intensity change and others based on a Fabry-Perot interferometer. Antunes et al. [27] reviewed fiber Bragg grating (FBG) based structural health monitoring techniques in recent years. Tennyson et al. [28] summarized the implementation of FBG-based structural health monitoring for innovative bridges in Canada. Moreover, Higuera et al. [29] discussed four successfully applied optical fiber sensor (OFS) technologies in SHM, and also summarized other useful fiber optic technologies for SHM. On this basis, a novel POFS for liquid level measurement is discussed here. The setup of the proposed sensor is presented in Figure 2. This sensor is installed in the cylinder and connected to the system, and deflection can be obtained by the liquid height variation inside the cylinders when the bridge structure deforms.

The proposed POFS contains a floating block, two polytetrafluoroethylene (PTFE) tubes, and plastic optical fibers. The working principle of the sensor is based on the Archimedes principle. The floating block is suspended by its buoyancy. When the structure deforms, the distance between the top of the cylinders and the liquid level varies accordingly. Therefore, the positions of the floating blocks in the cylinders will be changed due to buoyancy, reflecting the deflection of corresponding points of the structure. The polytetrafluoroethylene (PTFE) tubes and plastic optical fibers then transfer the deflection to the intensity change in the optical fiber. As Figure 2 shows, *d* is the magnitude of the gap between the separated fibers that equals the distance variation between the floating block and the top of the cylinder. Thus, by measuring *d*, the bridge deflection can be obtained, which the proposed POFS was designed for. The cylinder is plastic, and a rigid base is designed for this cylinder to make this cylinder easy to install at selected points of the structures. The internal suspended body is composed of cystosepiment and its density is apparently lower than the liquid, meaning it will always float on top of the liquid.

**Figure 3:**
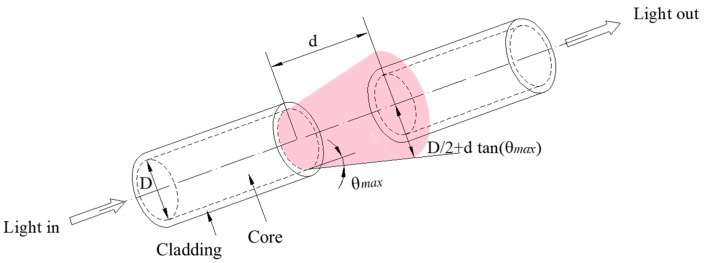
The schematic diagram of the sensing principle.

The basic principle of the proposed POFS mainly depends on the light intensity modulation with the distance change between the two cleaved plastic optical fibers. Here, the two cleaved glass fibers are secured by PTFE tubes to form the sensing gauge, and the separation of the cleaved ends varies. Hence, the light intensity in the circuit will be changed according to the distance in the tube when the structure deforms. The attachment of the PTFE tube to the cylinders, allowing displacement to be transferred to the sensor without loss, is critical. The relationship between *d* and intensity loss is well discussed in [30], and the operating principle is illustrated in Figure 3. The relative motion of the two-faced POFS renders the output light intensity variation. The relationship between the light intensity and the gaps of the POFS will be presented briefly as follows. Light emits from the source fiber, assuming that the energy distribution is homogeneous; its half-angle at the apex is denoted as *θ*_max_; *D* is the diameter of the fiber; *d* is the displacement to be measured; and the intensity loss is equivalent to the logarithm of the receiving fiber core area and the total area ratio, which can be given as:

(2) $$\eta =-10\log_{10}\left[\frac{{\left(\frac{D}{2}+d\tan\theta_{\max}\right)}^{2}}{{\left(\frac{D}{2}\right)}^{2}}\right]$$

To simplify Equation (2), this becomes:

(3) $$\eta =-10\log_{10}{\left(1+\frac{2d}{D}\tan\theta_{\max}\right)}^{2}$$

In addition, the stiffness and length of the PTFE tube may limit the amount of stretch, as well as the limited measuring range of the displacement. The measurement range also depends on the power of the light source. In this paper, a normal LED light source is applied for verification, its power is 20 mW, and the visible wavelength is 630 nm.

### 2.3. Manufacture of Liquid Level Sensing Element

**Figure 4:**
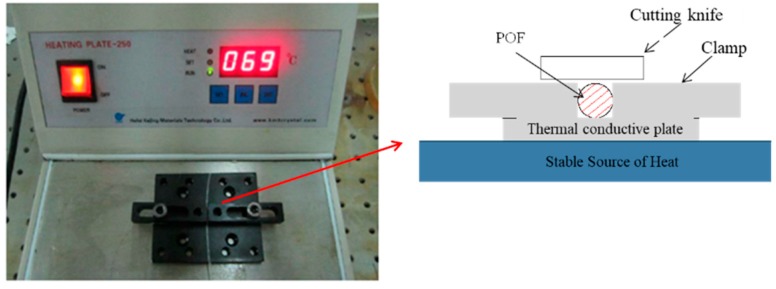
Equipment used for plastic optical fiber end cutting.

**Figure 5:**
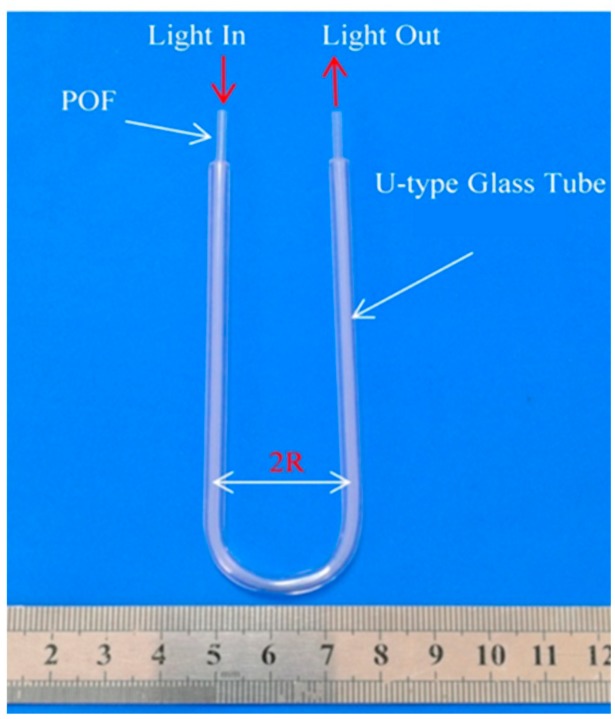
U-shaped plastic optical fiber fixing device.

The proposed sensor is low-cost, simple, and easy to be implemented. However, the quality of the cutting surface and U-fiber fixation are crucial for the working performance of the sensor. In this study, face machining was conducted by hot cutting and the ends were polished after cutting, as shown in Figure 4. The U-fiber fixation was handled by a U-shape glass tube, shown in Figure 5. 

## 3. Experimental Validation

To develop an advanced POFS-based connected pipe system, the plastic optical fibers can support this sensing technology to achieve better application. Compared to traditional sensors, the intensity-based POFs have more advantages, including immunity to electromagnetic interference, higher accuracy, smaller size, better anti-interference ability, and stability for long-distance transmission. As the sensing element is sensitive to the fiber diameter, bending radius of the U-shaped fiber, and the temperature variation, effective selection of the appropriate parameters for this element is difficult.

### 3.1. Setup for the Liquid Level Sensor Validation

**Figure 6:**
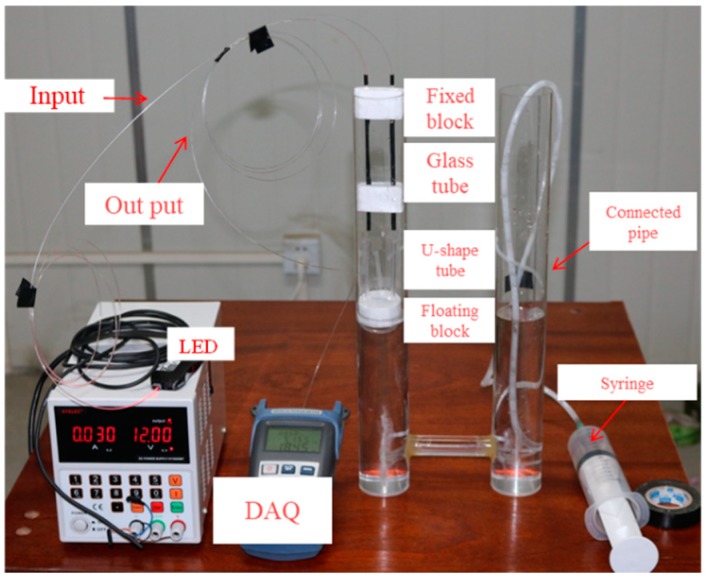
Experimental setup of a liquid level sensor. Note: DAQ = data acquisition; LED = light-emitting diode.

A prototype of the POFS is fabricated for validation purpose. The full range of the sensor is designed to be 20 mm and the resolution is about 0.1 mm. Figure 6 shows the experimental setup of the proposed prototype. The POFS is installed in the cylinder, and a syringe is used for liquid injection to make the liquid level of the container vary. In order to evaluate the performance of proposed POFS and assure the effectiveness of the POFS-based connected pipe system in bridge deflection monitoring, laboratory tests are further carried out. The linearity, accuracy, and repeatability of the POFS are evaluated, and the stability and environment effect are also considered in the lab tests. Two cylinders with proposed sensors are assembled. They are connected by acrylic pipes and filled with water in the setup. The level is controlled by the volume of the injected water.

**Figure 7:**
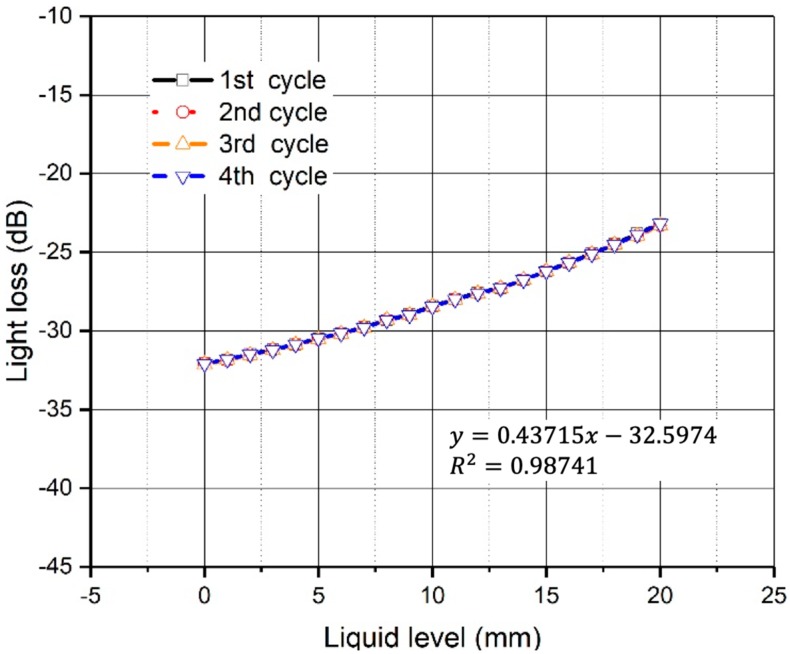
Short-term cyclic deflection test.

A series of slow varying liquid level cycles are conducted to the setup. These cycles result in the liquid level varying slowly from 0 to 20 mm, with a sub-step of 1.0 mm in 10 min. The liquid level starts from different points to assess the range and repeatability of the POFS. Its performance during the mentioned cycles conducted in invariable environments is shown in Figure 7. The correlation between the liquid level controlled by the syringe and the signal given by the variation of the light intensity measured in the plastic optical fiber is discussed. This shows the linearity across the whole range of the POFS, and the linear fit and $R^{2}$ are presented in the figure. According to the results, the resolution of the sensor is estimated as 0.1 mm by comparing the measured signal and the variation of the volume of liquid. The test results also indicate that the sensor shows good synchronization and excellent repeatability. 

During the tests, the full range of the proposed sensor is validated with different liquid levels. The maximum deviation of the sensor measurement is aroused when the liquid level variation is below 0.10 mm.

### 3.2. Structural Parameter Analysis for the Sensor

The performance of the proposed POFS is usually measured in terms of sensitivity and detection limit, which correspond to different core diameters of optical fibers and U-shaped fibers with different bending radii. Subsequent to the mechanical performance and characterization of the sensing element response, the environmental effect is analyzed.

(1) Core diameters

**Figure 8:**
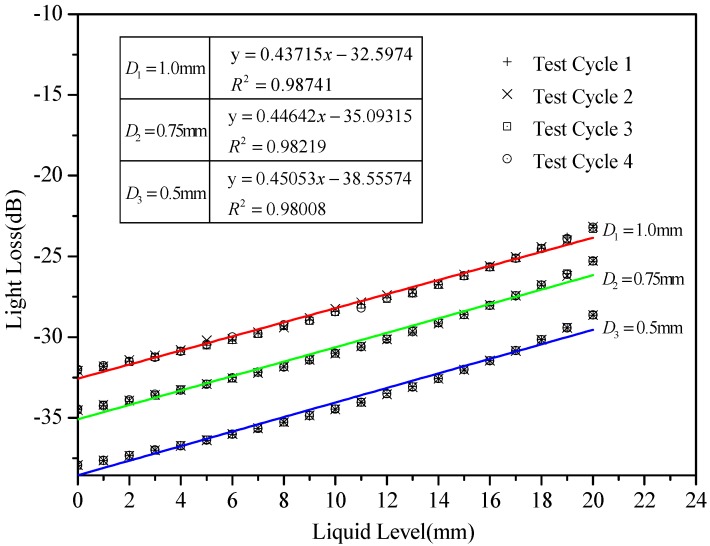
Plastic optical fiber sensor (POFS) performance test with different core diameters.

To investigate the effect of different core diameters on the measuring range and accuracy of the proposed POFS, sensors with fiber core dimensions of 0.5, 0.75, and 1.0 mm are tested, and the results are shown in Figure 8. The relationship between the water level and the intensity loss is fitted. Figure 8 also shows that sensors with small diameters are more sensitive than the one with a large diameter, which means that the sensitivity will be decreased when the core dimension is increased. However, for smaller diameter sensing elements, it is difficult to ensure the processing quality. In this paper, a sensor with a 1.0 mm core dimension fiber is finally employed in the following experiments.

(2) Bending radius

**Figure 9:**
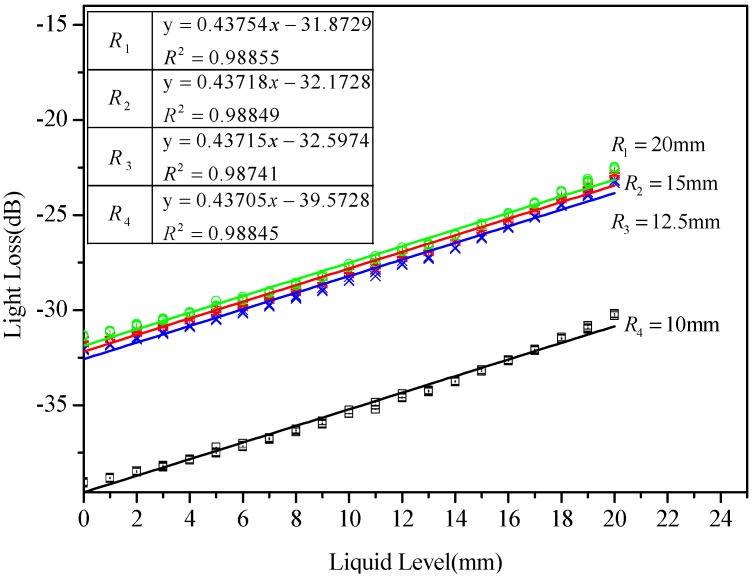
POFS performance test with different bending radii.

Considering the effect of the bending radius of the proposed POFS, the experiment was conducted to compare the sensitivity of the sensor with different U-shaped fiber bending radii of 10, 12.5, 15, and 20 mm. As shown in Figure 9, different lines illustrate the performance of the U-shaped sensing element with different bending radii. In the figure, the relationship between liquid level and intensity loss are presented, and the lines of the water level and intensity loss are fitted. According to the results, the intensity loss of the sensing element with the large bending radius is similar to the one with the small bending radius, which means that the influence of the bending radius is limited. Meanwhile, it reveals that the bending radius has a critical value, and when the bending radius of the U-shaped POFS is larger than it, the effect of the change of the bending radius is negligible and tends to be stabilized.

(3) Influence of the temperature

**Figure 10:**
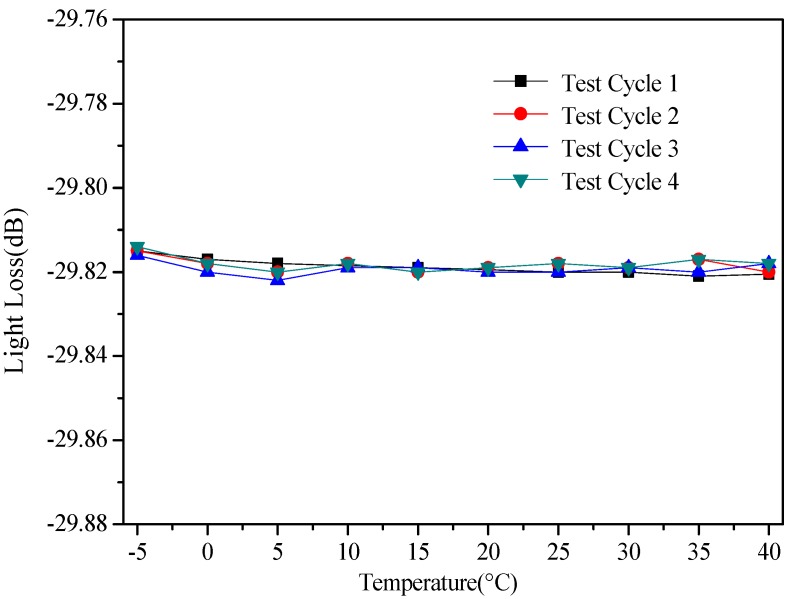
POFS performance test at different temperatures.

To develop an advanced POFS-based connected pipe system, the intensity-change-based POFS can support this sensing technology to achieve a better effect on the liquid level sensing element of this system. To investigate the temperature effect on the proposed POFS, an experiment is conducted in a small chamber, in which the temperatures varies from −5 °C to 40 °C. According to the results obtained from the test, as shown in Figure 10, the temperature variation has little effect on the proposed sensor. In the described conditions, the approximated periodic measurement error is less than 1%.

## 4. Experimental Study

### Experimental Setup

**Figure 11:**
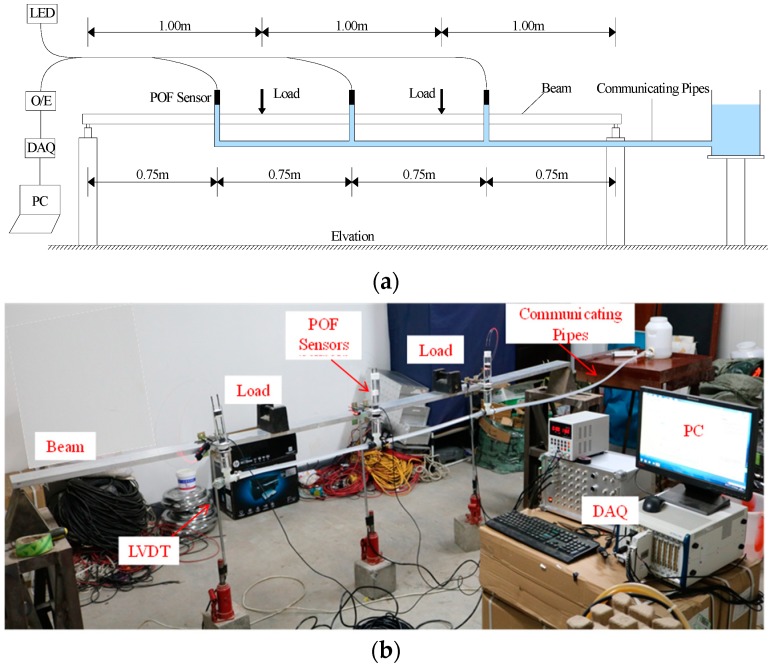
Experimental set-up of the deflection test: (**a**) the scheme for the employment of the POFS-based deflection monitoring system; (**b**) the installed system in the laboratory.

The experimental setup in this study is shown in Figure 11, which is composed of four components: connected pipes, plastic optical fiber liquid level sensors, data (obtained via data acquisition, DAQ), and a simple supported beam. The scheme for the use of multiple sensors is depicted in Figure 11a. Each sensor forms an independent channel together with its own light source and photodiode, and then the signal in each channel can be acquired by DAQ. In addition, the mentioned light source and photodiode are inexpensive and easy to purchase. Figure 11b is a photo of the installed system in the laboratory.

**Figure 12:**
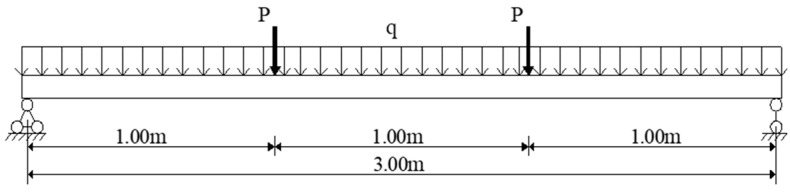
Load placement in the experiment.

**Figure 13:**
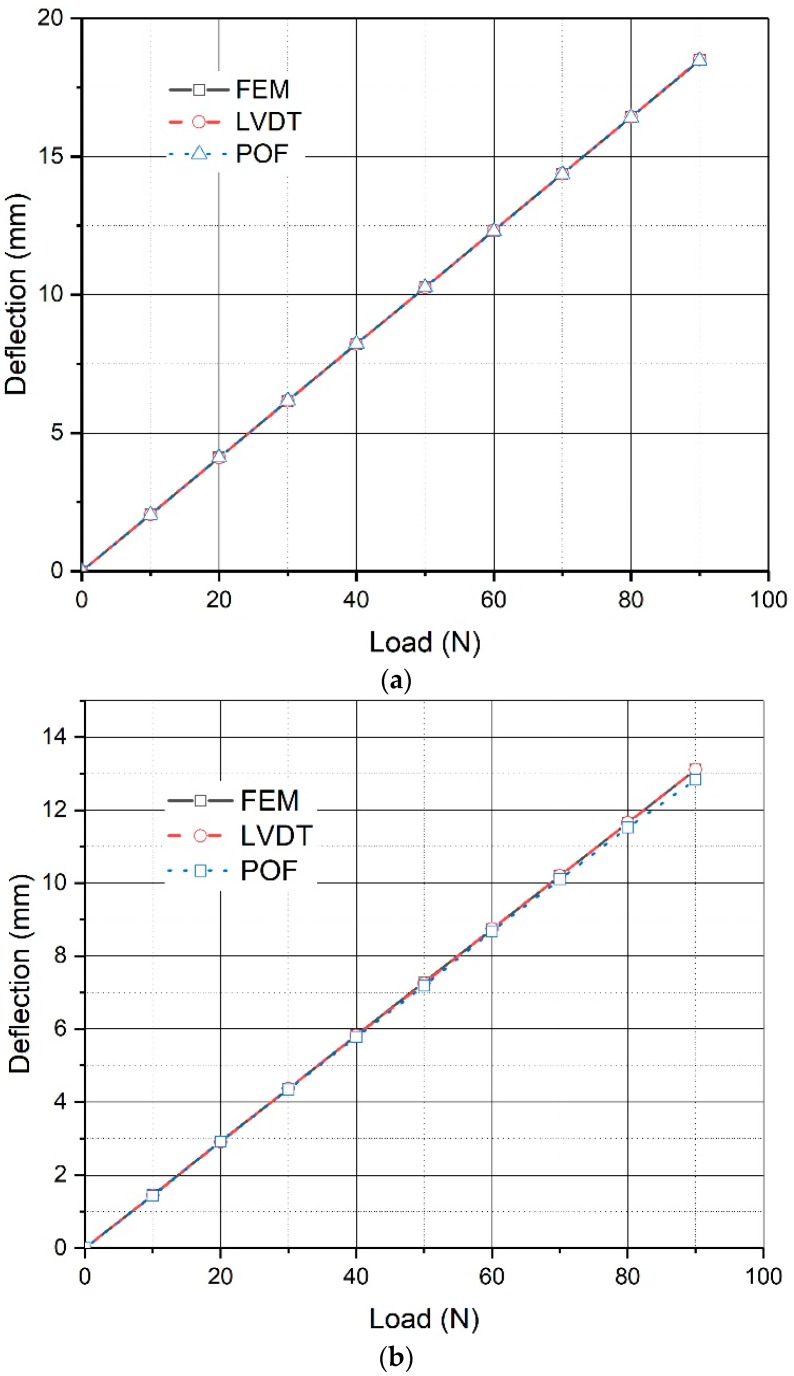
Comparison of the experiment results: (**a**) results at mid-span of the beam; (**b**) results at the quarter-span of the beam. Note: FEM = Finite element method.

A four-point bending test is employed, with the step-loading imposed on the simple supported beam increased from 10 to 90 kN, with a 10 kN increase at each step. Figure 12 shows the load placement of the static load test. The proposed deflection monitoring system is installed on the beam, and a water container is deployed at a fixed point outside the beam. Cylinders and containers are connected by acrylic pipes, and water is applied as liquid injected inside the system. Three LVDTs, whose measure error is less than 0.001 mm, are employed to measure the deflection of the beam. The original displacement data, measured after the weights are placed at specific locations, are compared with the LVDT results later. The results are presented in Figure 13.

The experimental results can provide useful information on the proposed system for real bridge applications. Figure 13 shows the displacements obtained in the quarter- and mid-span points of the beam during the load cycles. The results were achieved with sampling every 1 min, and each sample was obtained according to the average of 10 readings when the sampling rate was 1Hz. After this smoothing procedure, the residual perturbations resulting from the disturbance of the beam can be removed. In Figure 13, it is obvious that the displacements obtained in different sections of the simple supported beam during the load cycles were found to accord well with the numerical results and results of LVDTs, especially for the deflection of the mid-span. Regardless of whether the deflection increases or decreases, the proposed system always performs well when measuring the beam deflection.

## 5. Conclusions

In this paper, a POFS-based connected pipe system for bridge deflection measurement is proposed, and it is validated by a series of experiments. The developed system consists of a connected pipe and a novel plastic optical fiber liquid level sensor. The basic principle, fabrication, and characteristics of the proposed system are discussed in detail. Lab tests are conducted to reveal the linearity, accuracy, and stability of the developed system, and the deflection of the test beam is measured by the sensor, which agrees with LVTD within 0.1 mm. The effects of the environment on the proposed system are also discussed in this paper. Under conditions of varying temperatures, stable responses can be observed. The developed sensor presents a variation of 1.90% with the temperature varying from −5 to 40 °C and the sensibility of sensor is of the order of 0.44 dB/mm of displacement. The proposed system shows excellent potential to be a robust bridge deflection monitoring technology for structural health monitoring systems. 

The ability to build a distributed structural deflection monitoring network based on the proposed POFS-based connected pipe system without any fixed reference point in situ is a great advantage for civil structure monitoring, especially for long-span bridges. Some further works are still needed, for example exploring advanced sensor fabrication technologies and applications of novel materials for POFS in order to enhance the stability and sensitivity and reduce the cost of this type of monitoring technology.
